# Characterizing the likelihood of dengue emergence and detection in naïve populations

**DOI:** 10.1186/1756-3305-7-282

**Published:** 2014-06-23

**Authors:** Rebecca C Christofferson, Christopher N Mores, Helen J Wearing

**Affiliations:** 1 Department of Pathobiological Sciences, Skip Bertman Drive, School of Veterinary Medicine, Louisiana State University, Baton Rouge, LA 70803, USA; 2 Department of Biology, The University of New Mexico, Albuquerque, NM 87131-0001, USA; 3 Department of Mathematics & Statistics, The University of New Mexico, Albuquerque, NM 87131-0001, USA

**Keywords:** Dengue, Transmission, Arbovirus, Heterogeneity of infectiousness, Human infectiousness, Dengue introduction, Stochastic modeling, Dengue model, Viremia

## Abstract

**Background:**

Vector-borne disease transmission is dependent on the many nuances of the contact event between infectious and susceptible hosts. Virus acquisition from a viremic human to a susceptible mosquito is often assumed to be nearly perfect and almost always uniform across the infectious period. Dengue transmission models that have previously addressed variability in human to vector transmission dynamics do not account for the variation in infectiousness of a single individual, and subsequent infection of naïve mosquitoes. Understanding the contribution of this variability in human infectiousness is especially important in the context of introduction events where an infected individual carries the virus into a population of competent vectors. Furthermore, it could affect the ability to detect an epidemic (and the timing of detection) following introduction.

**Methods:**

We constructed a stochastic, compartmental model to describe the heterogeneity of human viremia and calculate the probability of a successful introduction, taking into account the viremia level (and thus acquisition potential) of the index case on, and after, the day of introduction into a susceptible population and varying contact rates between the human and mosquito populations. We then compared the results of this model with those generated by a simpler model that has the same average infectiousness but only a single infectious class.

**Results:**

We found that the infectivity of the index case as well as the contact rate affected the probability of emergence, but that contact rate had the most significant effect. We also found that the interaction between contact rate and the infectiousness of the index case affected the time to detection relative to the peak of the epidemic curve. Additionally, when compared to our model that accounts for variable infectiousness, a model with a single infectious class underestimates the probability of emergence and transmission intensity.

**Conclusion:**

Understanding the interplay between individual human heterogeneity of infectiousness and the rate of contact with the vector population will be important when predicting the likelihood, detection, and magnitude of an outbreak.

## Background

Disease transmission is dependent on the many nuances of the contact event between infectious and susceptible hosts. In the dengue (DENV) transmission system, we can explicitly define the contact events as 1) transmission from *Aedes* mosquitoes with a productive, disseminated DENV infection (where virus is present in the saliva and deposited upon mosquito probing and feeding); and 2) acquisition of virus by a naïve *Aedes* mosquito from an infected individual with systemically circulating DENV [1].

Heterogeneity in the first of these- transmission from *Aedes spp*. to humans- can be further subdivided into the process of dissemination and, ultimately, successful transmission. Vector competence is the inherent ability of a mosquito to support replication of a virus for subsequent transmission and, as a static measure, is often calculated as the total proportion of DENV exposed mosquitoes that develop a disseminated infection capable of transmission at a particular time point. Vector competence has been shown to vary based on viral and/or mosquito strains [2-7] as well as environmental and ecological conditions [2,4,8-11]. Further, there is variability of vector competence at the level of the individual mosquito, and as such, vector competence is best evaluated as a dynamic process through a mosquito population [7,12], which takes into account the extrinsic incubation period (EIP, the time it takes for an arbovirus to replicate sufficiently through the vector for transmission to a vertebrate host) as well as the mortality rate of the mosquito [2,10,11,13,14].

The second of these contact events, the acquisition of virus from a viremic human to a susceptible mosquito is often assumed to be nearly perfect and almost always uniform across the infectious period. DENV transmission models have previously addressed variability in human to vector transmission dynamics mostly in the context of antibody-dependent enhancement (ADE). These studies have focused on secondary (rarely, tertiary or later) infections and the theory that these infections have a higher intensity of transmission than primary infections due to ADE [15-19]. While these studies suggest that differential infectivity of DENV patients may be important, they are focused more on the average infectiousness of a group of individuals- severe versus not-severe DENV patients. These studies do not account for the variation in infectiousness of a single individual, and further, these studies do not directly address the consequences of this heterogeneity: the infection of naïve mosquitoes. Recently, a study in DENV infected patients showed that there is differential acquisition (and thus transmission) rates relative to individual host viremia among a cohort of symptomatic (both febrile and severe) individuals [20]. Understanding the contribution of this variability in human infectiousness is especially important in the context of introduction events where an infected individual carries the virus into a population of competent vectors. Since the introduction of DENV-1 into Florida in 2009-2010, the virus has become established in Key West and moved north into Miami and Martin county [21]. However, DENV has been detected in other locales in the United States such as Houston and Brownsville, TX, as well as Suffolk County, NY [22-25] without long-term establishment of the virus. This is an indication that our understanding of the factors involved in successful emergence of DENV remains limited.

While the factors affecting exposure to the virus are many (environment, seasonality, socioeconomic and lifestyle factors) [22,26], a critical component is the contact event between a viremic index case and the susceptible mosquito population. The key parameters affecting contact at the critical moment in introductory transmission into a completely susceptible population of mosquitoes are 1) the probability of acquisition of virus by a susceptible mosquito resulting from contact with the index case and 2) the contact rate between the mosquito and human populations. In addition, the interaction of these two transmission components defines the likelihood that an introduction will produce a fulminant, detectable outbreak.

Herein, we investigated the role of dynamic human infectiousness in determining the emergence potential and subsequent detection of DENV-1 into a naïve population of humans with the competent vector, *Ae. aegypti*. Additionally, while characterizing transmission explicitly in terms of the viremia curve and corresponding infectivity to mosquitoes, we determined how differential contact rates between the human and mosquito populations impacted the probability of detectable DENV-1 outbreaks.

## Methods

**Figure 1:**
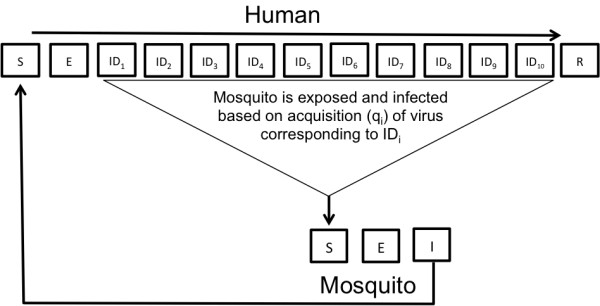
**Model schematic.** There were 10 human infectious classes each corresponding to a single infectious day (ID_1-10_). Mosquitoes exposed to DENV-1 infectious individuals are infected at a rate q_i_ associated with ID_i_.

**Figure 2:**
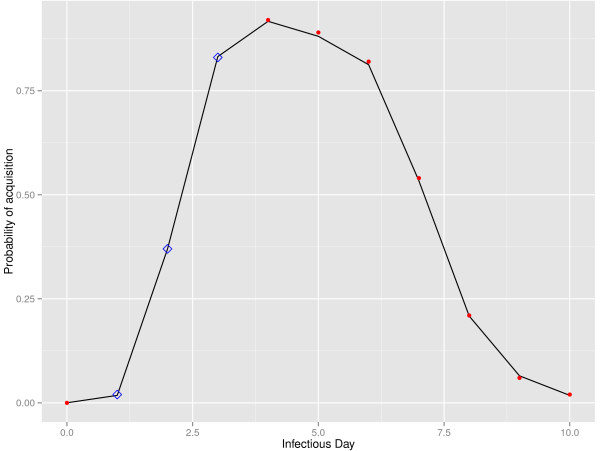
**Extrapolated fit of probability of acquisition.** The distribution of acquisition over infectious day (ID) given the relationship between infectious day and circulating viremia from [20]. Red dots are data points from [20], blue squares are the extrapolated points of acquisition for ID1-3 based on the curve fit (black line).

We constructed a stochastic event-driven model of DENV-1 transmission that explicitly accounts for heterogeneity of human viremia, based on the compartmental framework of Susceptible-Exposed-Infectious-Recovered classes for humans and Susceptible-Exposed-Infectious classes for mosquitoes (Figure 1). Variation in human viremia is modeled by dividing the human infectious period into subclasses, each representing a single infectious day (ID) with an associated level of infectivity. Data from Nguyen *et al.*[20] (DENV-1) are used to parameterize infectivity levels on a per day basis. Specifically, we derive the probability of acquisition given a specific level of viremia and the course of viremia over several days of illness (DOI) by extrapolating curves to the discrete values obtained from Figure 2 and Additional file 1: Figure S4 in [20], respectively. These data were compiled by allowing naïve *Ae. aegypti* mosquitoes to feed on viremic individuals whose level of viremia was later determined via qRT-PCR. Mosquitoes were incubated and later tested for the presence of virus and thus successful transmission [20]. Based on these data, we divide the human infectious period into 10 days in the model (classes ID_1_-ID_10_), with ID4-10 corresponding to the DOI2-8 from [20].

### Characterization of human viremia and mosquito acquisition potential

Our model assumes that viremia lasts roughly 7-10 days and viremia onset is, on average, 2 days prior to illness presentation, which is presumed to be DOI1 [20,27]. As the data from [20] spans DOI2-8, we wanted to extrapolate the viremia values of DOI1 and those two days prior to symptomology, with the assumption that three days prior to symptoms, the viremia was zero. We then define acquisition potential (q) as the probability that a naïve mosquito feeds on a viremic individual and subsequently acquires a viral infection from that blood meal. To determine the acquisition rates for the entirety of the infectious period, we did the following:

*1. Fit a function to the viremia curve from*[20]*with the assumption of zero three days prior to symptom onset*. We determined that the shape of the viremia curve was similar to that of a Weibull probability distribution function, but obviously the scale was not. Through an iterative process of linear transformation of the data (to reconcile the scale of viremia vs. a Weibull function), we minimized the maximum difference between the transformed data and the Weibull curve. Details are available in the Additional file 2. Goodness of fit compared to the original data was verified using a boot-strapped version of the two-sample Kolmogorov-Smirnov test (ks.boot procedure, R package Match), where the null hypothesis states there is no difference in the probability densities of the two data vectors. The p-value indicated this was the case (p-value > .9999) and the fit was determined to be sufficient.

2. *Estimated the logistic regression used to determine average acquisition given viremia level from Nguyen, et al.* Additional file 1: Figure S4 *and extrapolate to ID1-3*. We determined that the logistic fit had parameter scale = 0.75 and location = 6.5, and extrapolated values of acquisition of 2%, 37% and 83% for ID1, ID2, and ID3 respectively (Figure 2), (fitting performed using R package Mass). An iterative process was performed to minimize the maximum difference between the data and fitted values.

### DENV transmission model and experimental design

To investigate the role of human infectiousness in the context of a potential introduction event, we initialized the model with a single index case. With respect to the viremia curve from [20], we varied the point at which the index case was introduced to the susceptible mosquito population. Thus we ran iterations of the model for each of the following scenarios. Introduction during: the latent period (ID0) and then in each of the infectious classes ID1-10.

We simulated the resulting emergence (n = 2000 realizations) for each parameterization of the model and calculated the probability of successful emergence of DENV-1 following the introduction via index case. We defined a successful introduction relative to the detection of cases, which we based on a recent sero-survey from Key West, FL when it was determined that likely only 2% of DENV-1 cases presented clinically and were reported [21]. Thus, a successful outbreak may go undetected if the expected total number of DENV-1 cases was less than 50 (i.e. 2% < 1). Because detection is a chance event, a 2% clinical presentation rate may actually result in more or less than 50 cases. Here, to focus on the effects of heterogeneity of infectiousness and varying contact rates, we define a successful, detectable introduction resulting in emergence as one where there were at least 50 total cases resulting from the single index case. Given the recent introduction of DENV-1 in Martin County, FL in the summer of 2013 [28], we based our total and susceptible populations on the two main urban centers of that county, Palm Springs and Stuart Island, which have a combined total population of approximately 38,000 people. The rest of the county is largely rural and thus less likely to be ecologically compatible with the DENV-1 vector *Ae. aegypti*.

Given that transmission from humans to mosquitoes is ultimately tied to contact between the two populations, we investigated the role of contact rate (a) on the probability of a detectable epidemic. Thus, we evaluated our model over a range of average contact rates: twice daily (a = 2), once daily (a = 1), once every 1.3 days (a = 0.75), once every two days (a = 0.5), approximately once every 3 days (a = 0.33), and once every 4 days (a = 0.25).

#### Assumptions and parameterization

Our model was run for a single year, without explicitly accounting for spatial or temporal variability in model parameters. Given the short time of simulation relative to human lifespan, birth and death rates of the human population were ignored and a constant population size was assumed. For female mosquitoes, we assumed a constant emergence rate and a constant per mosquito mortality rate, which leads to a stable female mosquito population. Since our efforts were performed with sub-tropical South Florida in mind, we assumed that the weather was permissive for *Ae. aegypti* year-long and that the abundance of *Ae. aegypti* did not differ significantly among seasons [29].

The model is defined by the events and corresponding transition rates in Table 1, and the parameter values (with sources) are given in Table 2. Stochastic realizations of the model were simulated using an algorithm that implements the tau-leap approximation to Gillespie’s algorithm [30]. A time-step of 0.125 days was chosen to achieve computational efficiency without sacrificing accuracy. All calculations, curve fitting and model simulations were performed in R version 3.0.1. Stochastic simulation code is included as a Additional file 2 (SEI10R_stoch_model.R).

**Table 1:** Definition of transition rates between compartments of the stochastic S-E-I-R model

**Event**	**Change in state**	**Transition rate**
Transmission from mosquito to human	(S_h_,E_h_) → (S_h_-1, E_h_ + 1)	aS_h_(I_m_/N_h_)
Onset of infectiousness in human	(E_h_,ID_1_) → (E_h_-1, ID_1_ + 1)	z_e_E_h_
Transition from ID_1_ to ID_2_	(ID_1_,ID_2_) → (ID_1_-1, ID_2_ + 1)	v_1_ID_1_
Transition from ID_2_ to ID_3_	(ID_2_,ID_3_) → (ID_2_-1, ID_3_ + 1)	v_2_ID_2_
Transition from ID_3_ to ID_4_	(ID_3_,ID_4_) → (ID_3_-1, ID_4_ + 1)	v_3_ID_3_
Transition from ID_4_ to ID_5_	(ID_4_,ID_5_) → (ID_4_-1, ID_5_ + 1)	v_4_ID_4_
Transition from ID_5_ to ID_6_	(ID_5_,ID_6_) → (ID_5_-1, ID_6_ + 1)	v_5_ID_5_
Transition from ID_6_ to ID_7_	(ID_6_,ID_7_) → (ID_6_-1, ID_7_ + 1)	v_6_ID_6_
Transition from ID_7_ to ID_8_	(ID_7_,ID_8_) → (ID_7_-1, ID_8_ + 1)	v_7_ID_7_
Transition from ID_8_ to ID_9_	(ID_8_,ID_9_) → (ID_8_-1, ID_9_ + 1)	v_8_ID_8_
Transition from ID_9_ to ID_10_	(ID_9_,ID_10_) → (ID_9_-1, ID_10_ + 1)	v_9_ID_9_
Recovery in human	(ID_10_,R_h_) → (ID_10_-1, R_h_ + 1)	v_10ID10_
Adult (female) mosquito recruitment	(S_m_) → (S_m_ + 1)	ϵ_m_
Susceptible mosquito death	(S_m_) → (S_m_-1)	μS_m_
Transmission from human to mosquito	(S_m_,E_m_) → (S_m_-1, E_m_ + 1)	aS_m_ Σ_i_(q_i_ID_i_/N_h_)
Exposed mosquito death	(E_m_) → (E_m_-1)	μE_m_
Onset of infectiousness in mosquito	(E_m_,I_m_) → (E_m_-1, I_m_ + 1)	bE_m_
Infectious mosquito death	(I_m_) → (I_m_-1)	μI_m_

Events and corresponding transition rates in the stochastic SEI_10_R-SEI model. For each event, we list only those states that change. Variable definitions are given in the Additional file 2 and parameter definitions and values are given in Table 2.

**Table 2:** Values, definitions and sources for parameters used in modeling efforts

**Parameter (value)**	**Definition**	**Reference**
a	Contact rate	Varied (.25-2)
b^-1^ (9 days)	Average extrinsic incubation period	[14]
μ^-1^ (18 days)	Average mosquito lifespan	Approximated from [43-45]
ϵ_m_ (5000 females/week)	Emergence rate of adult mosquitoes	[31]
z_e_^-1^ (4 days)	Latent period (humans)	[27]
(v_1-10_)^-1^ (1 day each)	Duration of each infectious subclass (ID1-10)	[20]
q_1_ (.02)*	Acquisition potential	*Extrapolated or directly from [20]
q_2_ (.37)*		
q_3_ (.83)*		
q_4_ (.92)		
q_5_ (.89)		
q_6_ (.82)		
q_7_ (.54)		
q_8_ (.21)		
q_9_ (.06)		
q_10_ (.02)		

Note that the emergence rate (ϵ_m_) results in an average mosquito density (per person^-1^) of <0.5.

*Mosquito acquisition (q_1-3_) values were extrapolated by curve fitting while q_4-10_ were taken directly from Nyguen, et al. [20] as described.

## Results

### Probability of detectable epidemic: relative infectiousness and contact rate

**Figure 3:**
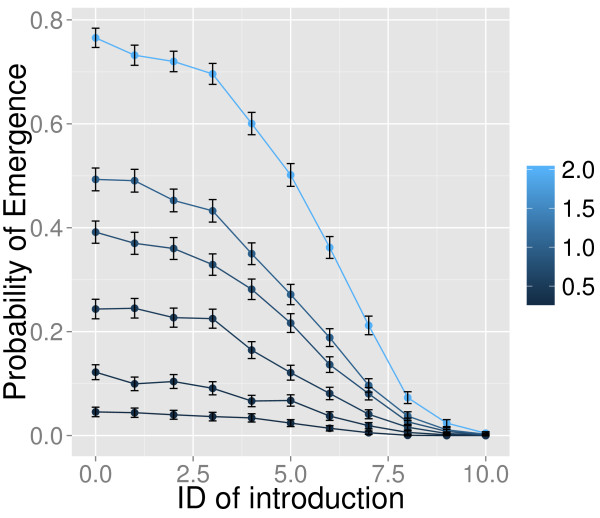
**Probability of successful, detectable emergence of DENV-1.** Probability of DENV-1 emergence (y-axis) given the introduction via an index case on infectious day (ID, x-axis) for several contact rates (a) (defined by the color bar on the right). Error bars correspond to 95% binomial confidence intervals:

We investigated how heterogeneity in infectivity during the course of a human infection affects detectable emergence potential of DENV-1 following introduction by an index case. Specifically, we varied the timing of introduction relative to the infectiousness of the index case when s/he enters the susceptible population of humans and in the presence of a competent vector population. The probability of emergence was expectedly higher on those days when acquisition potential was highest, and was minimal for ID9-10. (It is important to note that these are not cumulative probabilities, but based on the cumulative effect of introduction on a particular ID. That is, for ID1, the probability reported is the probability of a successful introduction given that the index case comes into the susceptible population at ID1, but transmits at the appropriate rates from ID1 until cessation of viremia.) Probabilities of emergence are depicted in Figure 3 and given in Additional file 1: Table S1.

$$p\pm 1.96\sqrt{p\left(1-p\right)n^{-1}}.$$

A daily contact rate of 2 produced a detectable outbreak 77% of the time if an index case was introduced into a naïve population during the exposed period (ID = 0). The probability remained relatively high (≥70%) for ID1-3, and then dropped to approximately 60%, 50%, 36%, 21%, 7%, 2% and <1% for ID4-10, respectively.

When the contact rate was once daily (a = 1), the risk of emergence was reduced to 49% for ID0 and >40% for both ID1-3. This probability did not drop off as steeply for ID4 and ID5, which had probabilities of 35% and 27%, respectively. The probability of emergence was then 19% for ID6, 10% for ID7, and 3.8% for ID8. Again, the probability of emergence was minimal (~1%) on ID9-10.

The risk of emergence for a = 0.75 was also very similar for ID0-ID4 (39%, 37%, 36% 33%, and 28%, respectively) and then dropped to 22%, 14%, 8%, and 3% for ID4-8, and then became ≤1% on ID9 and ID10. The probability of emergence when contact rate was once every two days (a = 0.5) was (in order of ID0-ID10) 24%, 24.5%, 23%, 17%, 12%, 8%, 4%, 1.6% and for ID9-10, <1%. For all days but ID0, the probability of emergence when a = 0.33 was consistently below 15% (range = [<1-12%]). For a = 0.25, the probability of emergence was consistently below 5% (range = [.1-4.5%]), for ID0-9 and 0 for ID10. For contact rates less than a = 0.5, transmission was either very tenuous (a = 0.25) or did not peak for all simulations within 365 days (a = 0.33).

Examination of the relationship between the probability of successful emergence and contact rate found that it followed an approximately negative exponential curve (Additional file 1: Figure S1).

### Window of Infectiousness

We were interested in determining the extent to which the length of an index case’s productive infectivity was altered due to differential contact rate. That is, at what contact rates are periods of especially low viremia able to result in a successful transmission event (from a human to a mosquito) and, conversely, at what point does the contact rate become such that only peak days of viremia contribute to the detectable emergence potential of DENV. The rate of contact between the index case and susceptible mosquito population altered the “window of infectiousness” of that index case. Indeed, the probability of emergence during the days of lowest acquisition potential (ID 8-10) was consistently less than 10%. For ID9 and ID10, the probability was even lower: at its highest only 2.4%, and otherwise ≤1.1%, meaning that periods of similarly low viremia do not contribute meaningfully to the emergence potential of DENV-1. In addition, with a contact rate of a = 0.25 the window of infectiousness was truncated by two days as ID9 and ID10 were non-productive and did not result in a successful emergence event.

### Proportion of population infected

The magnitude of resulting outbreaks during the time over which the simulations were realized (365 days) was also examined. When contact was equal to or greater than a = 0.5, there was no apparent effect of ID on the cumulative proportion of the population that became infected during those 365 days. For a = [0.75, 2], there was saturation of the population (>99%) (Additional file 1: Table S1). That is, almost all susceptible individuals became infected, assuming no other forces interrupted transmission (such as environmental phenomenon, etc.). At a contact rate of a = 0.5, the proportion of the population infected was approximately 97%, regardless of the day of introduction. At a = 0.33, the proportion of the population infected ranged from 47-67.5%; and at a = 0.25, the range was 1.5-.3.7%.

### Time to peak cases and transmission intensity

We were interested in the time between probable detection of a DENV-1 outbreak and the epidemic peak. To examine this, the peak number of cases was centered at time = 0 for each model realization that met the criteria for an outbreak (cumulative cases ≥50), and then the average number of cases for each time point relative to time = 0 was calculated (time = time_actual_– time_centered_).

**Figure 4:**
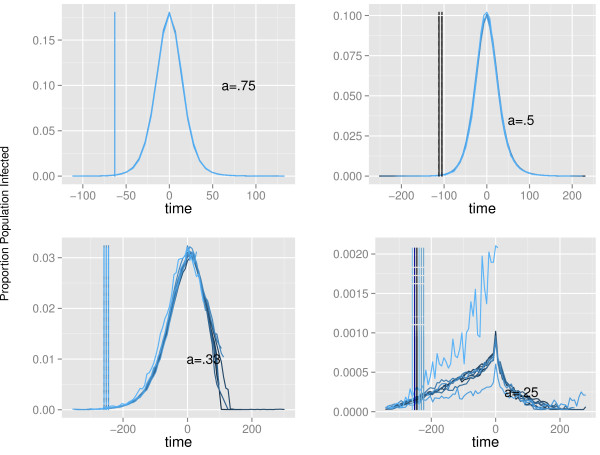
**Proportion of population infected.** The proportion of the population infected (y-axes) plotted against centered time (x-axes) for contact rates a = [.25-.75] and each day of introduction relative to the index case infectious day (ID). Vertical lines indicate the time of detection relative to peak cases at t = 0.

We assumed that there needed to be 50 cases before a clinical case would be detected, given the subclinical rate we extrapolated from the emergence of DENV-1 in Key West [21]. The time to peak cases (relative to detection) is then defined as the time between peak number of cases (at time_centered_) and likely detection of the first clinical case. We determined that contact rate was understandably tied to the average time to peak cases, but also that the variance in time to peak cases due to ID was related to contact rate. When the contact rate was greater than a = 0.25, outbreaks were well defined and the time between detection and peak was relatively stable (Figure 4, Additional file 1: Table S1). However, when a = 0.25, epidemics were erratic and thus this metric of detection relative to peak timing was deemed uninformative (Figure 4, Additional file 1: Table S1).

A shorter time between detection and peak indicates either an epidemic where the preliminary outbreak is more intense in nature (versus a slow burn-in), or else is an artifact of an already tenuous transmission system.

To demonstrate this, we also investigated the cumulative proportion of the population that was infected at (or before) the peak of the epidemic. At the highest contact rate, the time to peak relative to detection was 4 weeks and the proportion of the population infected was between 65 and 69%. When the timing of the peak relative to detection was still relatively short (7-8 weeks for a = 1 and a = 0.75, respectively), the average proportion infected was approximately 60% at peak (range 58.6-64.1%). When the time to peak was between 15 and 16 weeks (a = 0.5), the proportion infected at peak decreased to approximately 52%. And at a = 0.33, when peak timing was between 35 and 37 weeks, the proportion infected at peak was between 33 and 40%. The proportion infected at peak for a = 0.25 ranged between 1 and 4%, further illustrating the tenuous nature of transmission at this contact rate. However, for the other contact rates, the inverse relationship between time to peak (relative to detection) and the average proportion of the population that had been infected suggests there is a relationship between time to peak from detection and transmission intensity.

#### Comparison with simpler SEI_1_R model

We compared the results of our SEI_10_Rmodel structure to those of a more traditional SEI_1_R model, which has a single infectious class with an average duration of 10 days. We parameterized acquisition as the average of the rates for each of the 10 days in our multi-class model ($\bar{q}$ =0.468). This was done to enable a more direct comparison of the models because the reproductive number of each model would be identical (see Additional files 1 and 2). Results of the simpler model are limited to predictions based on the exposed and single infectious period (equivalent to ID0 and ID1 above). Because introductions on ID0 and ID1 gave similar results, we focus on comparisons between the introduction events occurring during the latent period (ID0). When considering the probability of emergence, the simple model predictions were consistently below those of the SEI_10_R for contact rates higher than a = 0.25, and the difference in emergence probability ranged from 2.25% (a = 0.33) up to 16.3% (a = 2). For a = 0.25, the simple model overestimated the probability of emergence by 0.15%, but this is again likely an artifact of tenuous transmission over the time period of simulation (365 days). Results from this model are given in Additional file 1: Table S2, Figures S4 and S5.

Although the two models had near identical results when estimating the cumulative proportion of infected individuals, there were also differences in the timing of the peak relative to detection. The simple SEI_1_R model predicted a longer time to epidemic peak (relative to detection) when contact rates were sufficient such that the epidemic had always peaked during the 365 days: the disparity in timing estimates differed by +1 week (a = 2,1), +2 weeks (a = 0.75), and +3 weeks (a = 0.5). The SEI_1_R model also predicted time to peak relative to detection would be longer by one week when a = 0.33, though the outbreaks at this contact rate did not all peak within 365 days and such a temporal metric is likely skewed in this case. The differences in predictions between the two model formulations could have significant implications for control strategies, which are usually reactionary, but carefully considered due to resource constraints.

## Discussion

Our results highlight the importance of accounting for heterogeneity at the interface of human to vector DENV transmission. Our model framework has the granularity of day-to-day contribution of variable human infectiousness and quantifies how the infectious day of an introduced case can impact outbreak potential. Indeed, there have been many failed DENV introductions into South Florida [28], which may be due, in part, to travelers being in the latter stages of viremia. While virus was detected in these travelers [28], our model illustrates a possible reason as to why these individuals did not produce viable and persistent chains of transmission. In addition, accounting for heterogeneity in human infectiousness leads to more accurate predictions of emergence potential and peak timing compared to a model assuming only a single infectious class with the same average acquisition rate. In particular, for high contact rates, the simple model consistently underestimated the emergence potential and overestimated the time to peak cases from detection.

The magnitude of the effect of variation in human infectiousness is significantly modified by human-vector contact rates. Differences in contact rates can be indicative of several broad factors: mosquito behavior differences, ecological and environmental stressors, and mosquito avoidance behavior by people [32]. Given the distribution of the primary vector *(Ae. aegypti*) as well as a competent alternative vector (*Ae. albopictus*), not only in tropical and sub-tropical regions but in urban centers like NYC and Atlanta [33], reducing human-mosquito contact is likely the only feasible means of transmission interruption. This can be achieved by several means: 1) insecticide-based vector control; 2) breeding source reduction; and 3) improved infrastructure in areas of lower socio-economic status (e.g., window/door screens). The recent DENV-1 emergence in Martin County was in a more established neighborhood where people were anecdotally reported to have increased contact with the mosquito population due to extended periods spent outdoors and the absence of infrastructure (such as screens and air conditioning) [34].

**Figure 5:**
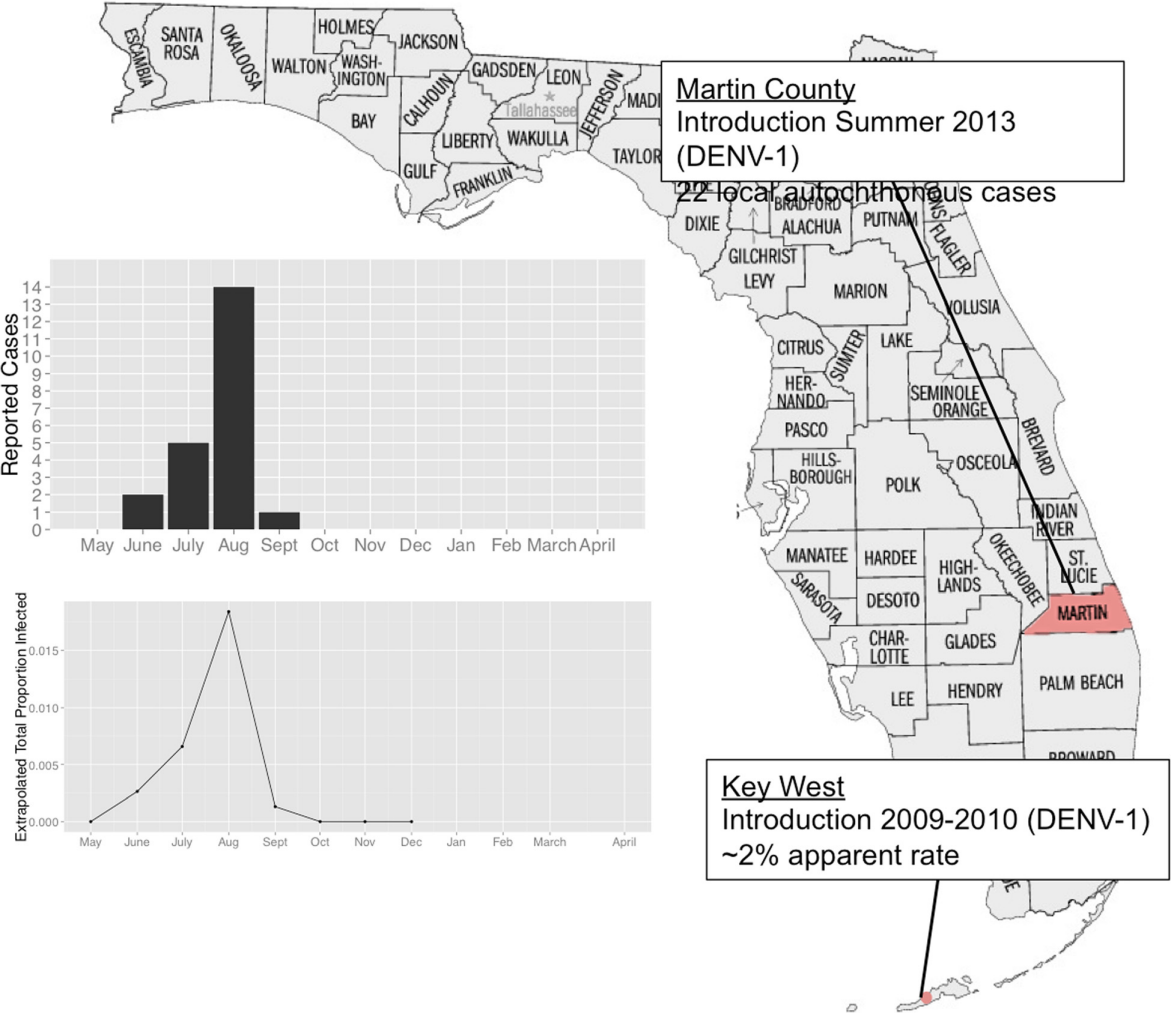
**Recent DENV-1 emergence in South Florida.** Map of Florida showing Key West where DENV-1 was introduced in 2009-2010 and Martin County, which experienced a DENV-1 introduction and emergence in summer 2013. The bar graph depicts the number of reported cases from Martin County and the line graph depicts the extrapolated total proportion of residents infected using the sero-conversation rate of Key West.

In fact, Martin County, FL had 23 cases of autochthonous DENV-1 transmission, 15 of which were residents of the county, in the summer of 2013, beginning in July and lasting through late September (approximately 10-12 weeks) [28]. These cases were all reported to the Florida Department of Health, and were clinically presenting individuals. Assuming that the rate of clinical presentation of DENV-1 in Martin county would be similar to that of Key West following DENV-1 introduction there (~2%) [21], then Figure 5 shows the extrapolated upper limit of total (cumulative) cases (clinical + subclinical = 1100 cases ~ 2.9% of the likely at-risk population). Our simulations suggest that, given the relative environmental stability of the area for the duration of the epidemic and forgiving other large-scale extrinsic forces, the average daily contact rate across the at-risk population (Palm City and Stuart Island) is more likely to have been closer to 0.25 than higher values. However, as DENV is often associated with clusters, it is likely that 1) the spatial scale of the epidemic was more limited, 2) the at-risk population was smaller and 3) the contact rate likely higher within that population [34-36].

### Caveats

There are a few model assumptions that deserve further consideration. First, we consider only the primary DENV vector, *Ae. Aegypti,* although *Ae. albopictus* is a competent vector for DENV, feeds on humans, and is present in the United States [29,37]. A major motivation of this was that the data on which the acquisition fit and subsequent modeling was performed comes from a human-*Ae. aegypti* experiment [20]. While no direct acquisition studies involving *Ae. albopictus* have been published, there are important differences in the vector competence of this secondary vector [38]. However, parameterization of this model could easily accommodate an effort focused on *Ae. albopictus* with appropriate attribution given to foraging preference, contact rates, and ecological niche of this species [39]. In terms of a two-mosquito model, that is the focus of on-going work in our laboratory and out of the scope of this particular effort.

Second, our assumption regarding presentation and detection rate (2%) is based on the Key West, FL experience [21] because Key West represents the most likely scenario to be repeated on subsequent introduction: 1) a mostly naïve population, so the risk of secondary DENV is less than endemic areas, 2) relatedly, the clinical presentation rate (associated with secondary infections) will be more similar than in DENV-endemic areas and 3) endemic areas are often set up for active disease surveillance and the medical community is trained to look for DENV symptoms. As DENV is a newly emergent pathogen in the United States, our surveillance and reporting infrastructure is still developing and thus best based on the most recent event: Key West, FL in 2009.

Finally, future consideration should be given to viremia differences within and among serotypes of DENV, as evidenced by the experimental data in Nguyen *et al.*[20]. Another study has also reported differences among serotypes as well as an approximate log difference in DENV-1 average viremia between primary and secondary infections [40]. While secondary infections would not be relevant for emergence of DENV, it would be very relevant for prediction of the incursion of a second serotype into an area that has only experienced one. This is likely going to be relevant in the near future as DENV-1 has been involved in autochthonous transmission in at least two counties in Florida, but other serotypes are often introduced by travelers [28].

## Conclusions

DENV has been purported to be a pathogen spread primarily to new areas by people, rather than its vector, which has a relatively short flight range [41-43]. The relative infectiousness of an individual is especially important when that individual is a potential index case, introducing the pathogen to a new area. In the case of DENV and other vector-borne diseases, the important parameters are the infectiousness of the viremic individual to a naïve, competent vector and the contact between the infectious individual and a competent, sufficiently large mosquito population. In this paper, to quantify the emergence potential of vector-borne pathogens such as DENV-1 in predominantly susceptible populations, we incorporate a critical parameter (acquisition potential) that accounts for the viremic state of the index case. Further, by assessing differential contact, we address the role of other factors (like the suitability of the ecology that such an index case might inhabit or arrive in) in facilitating transmission and emergence. We observe that the probability of a successful introduction is greatly affected by the timing of introduction relative to the infectiousness of the index case and that when this heterogeneity is ignored, estimates of emergence probabilities are underestimated. Further, the contact rate between the human and mosquito populations contributes to the emergence potential and the intensity of transmission. In general, our results highlight the importance of data-driven parameterization of acquisition based on the dynamic viremia process, and accounting for transmission heterogeneity in terms of contact.

## Competing interests

The authors declare that they have no competing interests.

## Authors’ contributions

RCC, CNM and HJW conceptualized the model. RCC and HJW realized the model and analyzed the output. All authors contributed to the interpretation of the results and the preparation of the manuscript. All authors read and approved the final version of the manuscript.

## Supplementary Material

Additional file 1 **List of Variable Definitions and starting conditions for the model as well as details regarding how we fit the data from [**20**] to incorporate into our model. Figure S1A.** Viremia data and extrapolation. Viremia data from Nguyen, *et al*. (red points) and the extrapolation of viremia at ID13 (blue squares) based on the fitted curve (black line). **Figure S1B. **Acquisition data and extrapolation. Acquisition data from Nguyen, *et al*. (red points) and the extrapolation to viremia points extrapolated in Additional file 1: Figure S1A (blue squares) based on the fitted curve (black line). **Table S1.** Probabilities of emergence and detection of DENV-1, Average proportion of population infected and Average time between detection and peak number of cases. Figure S2. The proportion of the population infected (y-axes) plotted against centered time (x-axes) for contact rates a = [1,2] and each day of introduction relative to the index case infectious day (ID) (indicated by colour bar, but corresponding curves largely overlap). Vertical lines indicate the time of detection relative to peak cases at t = 0. **Figure S3.** Non-linear relationship between contact rate (a) and the probability of emergence of DENV-1. R0 calculations for SEI_10_R and SEI_1_R. **Table S2.** Results of SEI_1_R model. Figure S4. Probability of emergence given by the simple model structure of SEI_1_R (compare to in-text Figure 3). **Figure S5.** Epidemic curves from the simple SIE_1_R model (compare to in-text Figure 4 and Additional file 1: Figure S2). Click here for file

Additional file 2 R code of the SEI10R stochastic model with a set of starting conditions, parameter definitions, and function call. Click here for file
