# Supplementary material for: Characterizing the likelihood of dengue emergence and detection in naïve populations
Source: Parasit Vectors. 2014 Jun 23;7:282. doi: 10.1186/1756-3305-7-282 (PMC4082489; doi:10.1186/1756-3305-7-282)
Supplement: Additional file 1 — List of Variable Definitions and starting conditions for the model as well as details regarding how we fit the data from [20] to incorporate into our model. Figure S1A. Viremia data and extrapolation. Viremia data from Nguyen, et al. (red points) and the extrapolation of viremia at ID13 (blue squares) based on the fitted curve (black line). Figure S1B. Acquisition data and extrapolation. Acquisition data from Nguyen, et al. (red points) and the extrapolation to viremia points extrapolated in Additional file 1: Figure S1A (blue squares) based on the fitted curve (black line). Table S1. Probabilities of emergence and detection of DENV-1, Average proportion of population infected and Average time between detection and peak number of cases. Figure S2. The proportion of the population infected (y-axes) plotted against centered time (x-axes) for contact rates a = [1,2] and each day of introduction relative to the index case infectious day (ID) (indicated by colour bar, but corresponding curves largely overlap). Vertical lines indicate the time of detection relative to peak cases at t = 0. Figure S3. Non-linear relationship between contact rate (a) and the probability of emergence of DENV-1. R0 calculations for SEI10R and SEI1R. Table S2. Results of SEI1R model. Figure S4. Probability of emergence given by the simple model structure of SEI1R (compare to in-text Figure 3). Figure S5. Epidemic curves from the simple SIE1R model (compare to in-text Figure 4 and Additional file 1: Figure S2). [file 1756-3305-7-282-S1.pdf]

Supplemental Information

Variable Definitions:

Sh	Number of susceptible humans
Eh	Number of exposed humans (ID0)
I_1	Number of humans in infectious class 1 (ID1)
I_2	Number of humans in infectious class 2 (ID2)
I_3	Number of humans in infectious class 3 (ID3)
I_4	Number of humans in infectious class 4 (ID4)
I_5	Number of humans in infectious class 5 (ID5)
I_6	Number of humans in infectious class 6 (ID6)
I_7	Number of humans in infectious class 7 (ID7)
I_8	Number of humans in infectious class 8 (ID8)
I_9	Number of humans in infectious class 9 (ID9)
I_{10}	Number of humans in infectious class 10 (ID10)
Nh	Total number of humans
Sm	Number of susceptible mosquitoes
Em	Number of exposed mosquitoes
Im	Number of infectious mosquitoes

Initial Conditions:
Sh = 38,000; Eh, $I_{1\text{-}10}$ = 1 or 0; Rh=0; Sm=7500; Em=0; Im=0

Viremia Curve Fitting and extrapolation of missing points:
The Weibull density is given by:

$$f(x) = \frac{k}{\lambda}\left(\frac{x}{\lambda}\right)^{k-1} e^{-\left(\frac{x}{\lambda}\right)^k}$$

Let the viremia data from [20] be v_{data}, and the curve fit be v_{fit}. Then:

v_{fit} ~ Weibull (k=6.4, λ=1.9) and $(v_{fit} * 65) = v_{data}$ (Figure S1A)

Acquisition curve fitting and extrapolation of missing points:
Let the acquisition values extrapolated from [20] but based on the viremia data above be

q_{data} and the curve fit be q_{fit}. Then:

q_{fit}~ logistic (μ=6.5, σ=0.75) (Figure S1B)

Where the logistic distribution function is given by:

$$F(x) = \frac{1}{(1 + e^{\left(-\frac{x-\mu}{\sigma}\right)})}$$

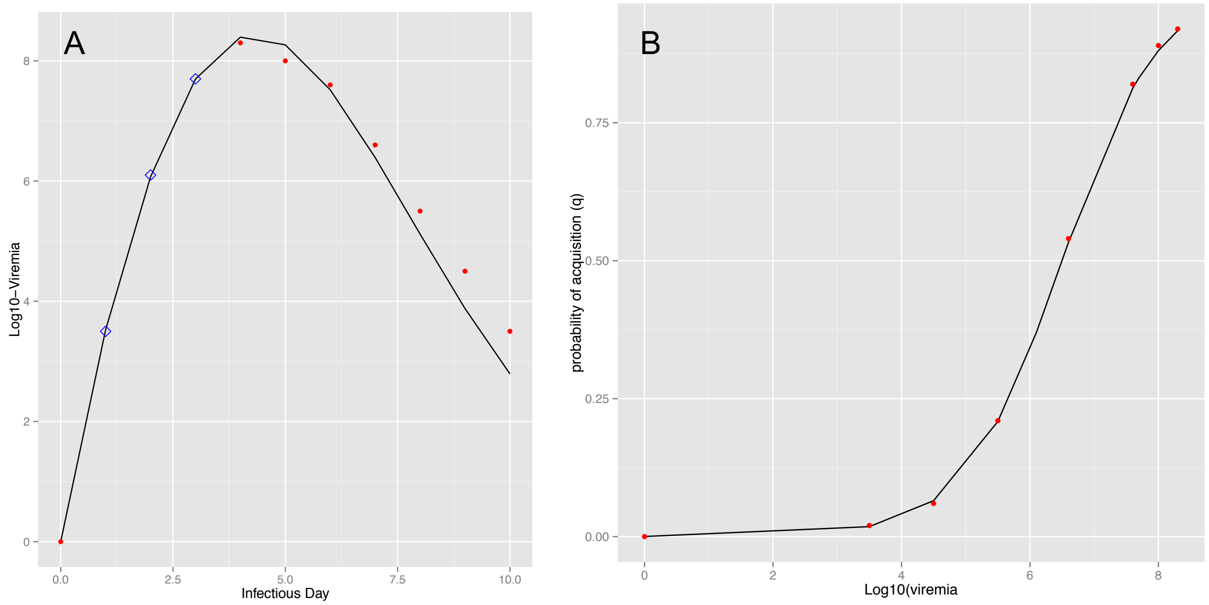

Figure S1A: Viremia data and extrapolation: Viremia data from Nguyen, et al. (red points) and the extrapolation of viremia at ID1-3 (blue squares) based on the fitted curve (black line). **S1B: Acquisition data and extrapolation:** Acquisition data from Nguyen, et al. (red points) and the extrapolation to viremia points extrapolated in Figure S1A (blue squares) based on the fitted curve (black line).

Table S1: Probabilities of emergence and detection of DENV-1, Average proportion of population infected and Average time between detection and peak number of cases: For each contact rate and infectious day (ID) of the index case upon introduction into a naïve population this table lists the probabilities of emergence and detection (cases ≥ 50), the average cumulative proportion of the population infected (after 365 days and at peak) and the average time (in days) between the detection of DENV-1 (cases ≥50) and the peak of the epidemic curve.

Daily contact rate (a)	ID	Probability of emergence	Cumulative % infected population	Cumulative % Infected at peak	Time to peak relative to detection (in days)
2	0	76.55%	>99	68.6	28
2	1	73.20%	>99	67.9	28
2	2	72.00%	>99	68.6	28
2	3	69.60%	>99	68.1	28
2	4	60.05%	>99	68.2	28
2	5	50.15%	>99	68.3	28
2	6	36.20%	>99	67.8	28

2	7	21.20%	>99	68.7	28
2	8	7.30%	>99	69.3	28
2	9	2.40%	>99	66.3	28
2	10	0.45%	>99	64.9	28
1	0	49.30%	>99	62.3	49
1	1	49.05%	>99	62.5	49
1	2	45.25%	>99	62.5	49
1	3	43.25%	>99	63.2	49
1	4	35.00%	>99	62.4	49
1	5	27.15%	>99	62.7	49
1	6	18.85%	>99	62.7	49
1	7	9.65%	>99	62.3	49
1	8	3.75%	>99	63.3	49
1	9	1.10%	>99	62.6	49
1	10	0.25%	>99	64.1	49
0.75	0	39.15%	>99	58.93748843	63
0.75	1	37.00%	>99	59.15036224	63
0.75	2	36.00%	>99	59.23561028	63
0.75	3	32.90%	>99	59.36390577	63
0.75	4	28.15%	>99	59.36891941	63
0.75	5	21.65%	>99	59.48033428	63
0.75	6	13.65%	>99	59.52627398	63
0.75	7	8.05%	>99	59.58290554	63
0.75	8	2.65%	>99	59.68093347	63
0.75	9	0.80%	>99	59.86190476	63
0.75	10	0.10%	>99	58.6	63
0.5	0	24.35%	96.8	52.3585308	105
0.5	1	24.50%	96.8	52.66330599	105
0.5	2	22.70%	96.8	52.38901716	112
0.5	3	22.50%	96.8	52.32870504	112
0.5	4	16.45%	96.8	52.31671742	112
0.5	5	12.10%	96.8	52.67823959	105
0.5	6	8.10%	96.8	52.56668191	105
0.5	7	4.10%	96.8	52.42643724	105
0.5	8	1.60%	96.8	52.85283813	112
0.5	9	0.40%	96.8	54.96527256	112
0.5	10	0.05%	96.8	53.15263158	105
0.33	0	12.20%	63.62013158	34.98331579	245
0.33	1	9.95%	63.71815789	34.91186842	252
0.33	2	10.40%	67.53331579	34.00894737	245
0.33	3	9.10%	61.73278947	32.90463158	252

0.33	4	6.65%	61.39044737	34.30913158	252
0.33	5	6.75%	63.11160526	34.35884211	245
0.33	6	3.75%	67.96513158	35.09852632	252
0.33	7	1.90%	70.73710526	36.54939474	252
0.33	8	0.60%	54.54055263	32.92621053	252
0.33	9	0.15%	47.8965	35.84386842	259
0.33	10	0.05%	59.21842105	40.56052632	252
0.25	0	4.55%	1.973216131	1.513332631	238
0.25	1	4.40%	2.283018538	1.647577307	245
0.25	2	4.00%	2.112758221	1.575638227	245
0.25	3	3.65%	2.257709519	1.640164223	252
0.25	4	3.40%	1.987136485	1.3712794	231
0.25	5	2.40%	2.191175044	1.717962188	245
0.25	6	1.40%	2.159831854	1.640957063	259
0.25	7	0.55%	1.469439508	0.880002278	224
0.25	8	0.05%	3.731578947	3.523684211	259

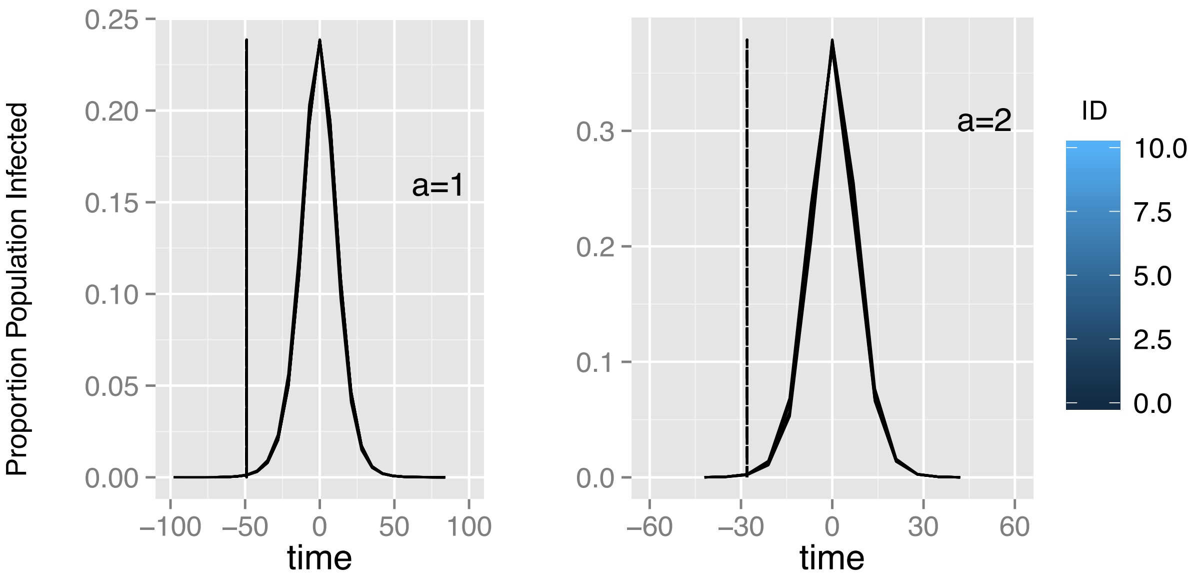

Figure S2: The proportion of the population infected (y-axes) plotted against centered time (x-axes) for contact rates a=[1,2] and each day of introduction relative to the index case infectious day (ID) (indicated by colorbar, but corresponding curves largely overlap). Vertical lines indicate the time of detection relative to peak cases at t=0.

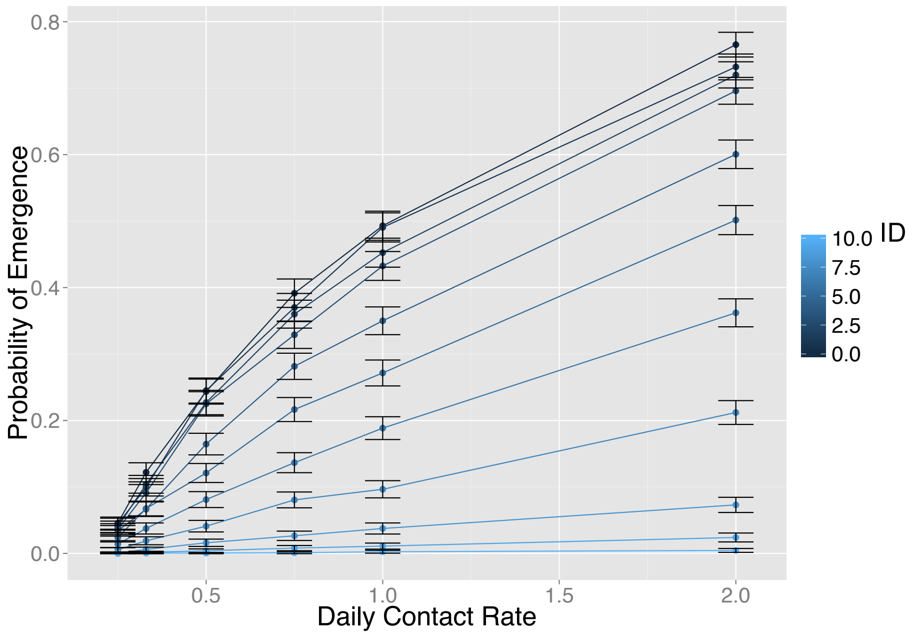

Figure S3: Nonlinear relationship between contact rate (a) and the probability of emergence of DENV1.

Basic Reproductive Number (R0) calculations for $SEI_{10}R$ and SEI_1R:

$SEI_{10}R$:

$$R0 = a^2\left(\frac{\varepsilon_m}{\mu N_h}\right)\frac{b}{(b+\mu)\mu}\sum_{i=1}^{10}\frac{q_i}{v_i}$$

SEI_1R:

$$R0 = a^2\left(\frac{\varepsilon_m}{\mu N_h}\right)\frac{b}{(b+\mu)\mu}\left(q_{avg} * 10\right)$$

where $q_{avg} = \frac{\sum q_i}{10}$.

Table S2: Results of SEI_1R model: The probability of emergence, cumulative % of population infected and % infected at peak, as well as time (in days) from detection to epidemic peak for the SEI_1R model are given below.

Daily contact rate (a)	Probability of emergence	Cumulative % infected population	Cumulative % infected at peak	Time to peak relative to detection (in days)
2	60.25%	>99	68.2	35
1	41.85%	>99	62.4	56
0.75	33.05%	>99	59	77
0.5	23.55%	97.2	52.5	126
0.33	9.95%	45.9	23.7	252
0.25	4.70%	1.3	0.88	n/a

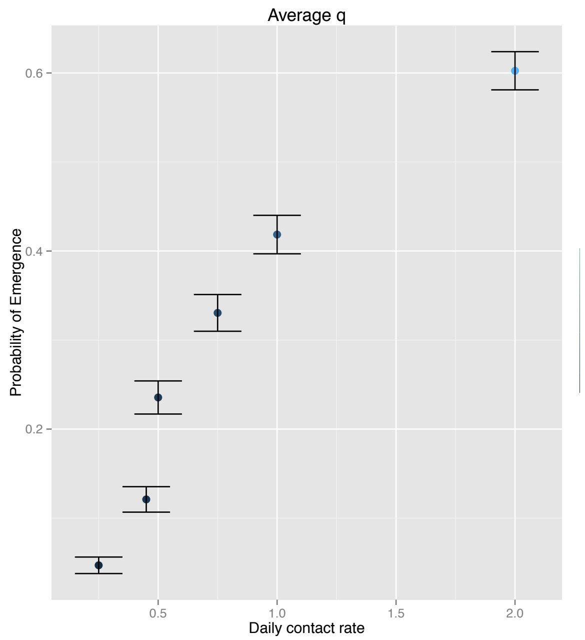

Figure S4: Probability of emergence given by the simple model structure of SEI_1R (compare to Figure S3).

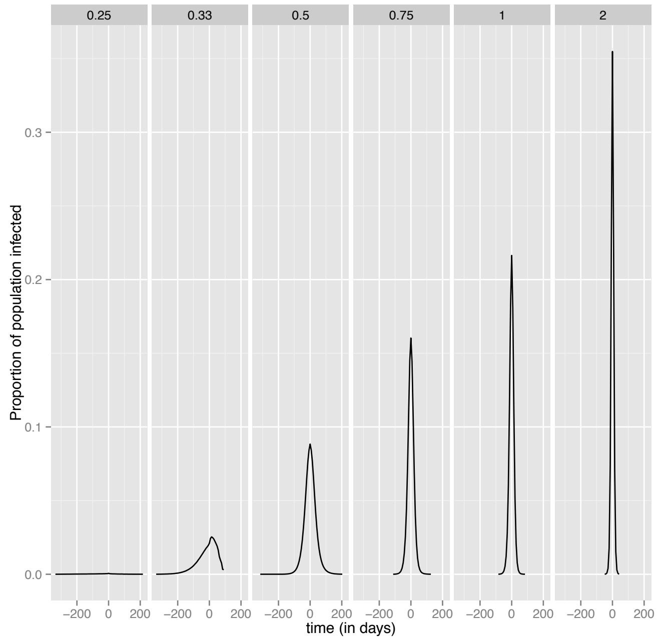

Figure S5: Epidemic curves from the simple SEI_1R model (compare to in-text Figure 4 and Figure S2).
